# Development of national consensus-based recommendations for history taking, examination and clinical management of infants in chiropractic practice

**DOI:** 10.1186/s12998-026-00671-x

**Published:** 2026-08-22

**Authors:** Freja Gomez Overgaard, Lise Hestbæk, Elisabeth Davidson

**Affiliations:** 1 Medical Spinal Research Unit, Spine Centre of Southern Denmark, University Hospital of Southern Denmark, Sygehusvej 24, 6000 Kolding, Denmark; 2 Department of Regional Health Research, University of Southern Denmark, Odense, Denmark; 3 Department of Chiropractic, Faculty of Medicine, Health and Human Science, Macquarie University, Sydney, Australia; 4 Center for Muscle and Joint Health, Department of Sports Science and Clinical Biomechanics, University of Southern Denmark, Odense, Denmark; 5 The Chiropractic Knowledge Hub, Odense, Denmark; 6 Kiropraxis, Private, Private Chiropractic Pratice, Copenhagen, Denmark

**Keywords:** Chiropractic, Pediatrics, Infants, Clinical guidelines, History taking, Physical examination, Musculoskeletal assessment, Manual therapy

## Abstract

**Background:**

Chiropractors in many countries provide care for infants, yet few structured, evidence-informed recommendations exist to guide history taking, examination, and clinical management in this population. In Denmark, variation in practice and limited public understanding of chiropractic care for infants highlighted the need for national recommendations to support safe, consistent, and transparent care.

**Objective:**

To describe the development of national recommendations outlining the minimum requirements for history taking, examination, and clinical management considerations for infants aged 0–12 months in chiropractic practice.

**Methods:**

These recommendations were commissioned by the Danish Society of Chiropractic and initially developed in 2020, with an update in 2025. The manuscript was reported with consideration of the RIGHT Statement. Recommendation development was informed by a targeted literature review, a national knowledge-sharing workshop involving 71 chiropractors, expert review by a seven-member panel with extensive paediatric experience, and external stakeholder consultation. Feedback from each stage was incorporated through an iterative drafting process.

**Results:**

The recommendations describe minimum requirements for medical history taking, red flag screening, general and musculoskeletal examination, referral, and clinical management considerations for infants. Minimum and supplementary assessment components are presented in structured tables to support consistent clinical assessment and decision-making.

**Conclusions:**

These national recommendations establish minimum requirements for history taking, examination, and clinical management considerations for infants in chiropractic practice. Developed through a structured process integrating a targeted literature review, practice-based input, expert review, and stakeholder consultation, they provide a transparent and practical framework to support safe, consistent, and collaborative paediatric care. Transparent reporting of the development process may facilitate the adaptation of these recommendations and the development of similar recommendations in other healthcare settings.

## Background

This project was commissioned by the Danish Society of Chiropractic to develop national recommendations for the history taking, examination, and clinical management of infants aged 0–12 months in Danish chiropractic practice. These recommendations aim to support safe, consistent, and transparent clinical care while facilitating communication with parents and other healthcare professionals.

Infants represent a regular patient group in Danish chiropractic practice. In 2018, children younger than one year accounted for approximately 3.5% of all patients seen by Danish chiropractors, corresponding to around 13,000 consultations annually, although considerable variation exists between individual practitioners [1, 2].

The reasons for seeking chiropractic care are relatively consistent across Scandinavia and other Western countries. Danish and Norwegian studies have shown that infantile colic or excessive crying, asymmetrical movement patterns, musculoskeletal concerns, breastfeeding difficulties, and sleep disturbances are among the most common reasons parents seek chiropractic care for infants [1, 2]. Similar findings have been reported in Norway [3] and elsewhere in Europe [4], suggesting that the clinical presentations of infants in chiropractic practice are broadly comparable across healthcare systems.

Although these presenting complaints are common, many are non-specific and may reflect a broad spectrum of underlying conditions, ranging from benign musculoskeletal dysfunction to serious medical pathology [5]. Consequently, a thorough medical history and physical examination are essential to identify children requiring referral while ensuring that those appropriate for conservative care receive consistent assessment and management [5, 6].

Despite infants representing an important patient population in chiropractic practice, there is limited published guidance describing the minimum requirements for their assessment. Existing publications primarily describe patient characteristics and presenting complaints [1, 3, 4], broader best-practice recommendations for paediatric chiropractic care [6, 7], or evidence relating to individual treatment approaches [8–10]. However, practical recommendations specifically describing the minimum requirements for history taking, physical examination, recognition of red flags, referral, and subsequent clinical management of infants in chiropractic practice remain limited.

The present recommendations were therefore developed by integrating current evidence, structured practice-based input, expert review, and external stakeholder consultation. Transparent reporting of the development process may facilitate adaptation of similar recommendations in other countries and clinical settings where high-quality evidence remains limited and recommendations must therefore integrate research evidence with structured clinical expertise [11].

The aim of this project was to describe the development of national recommendations outlining the minimum requirements for history taking, examination, and clinical management considerations for infants (0–12 months) in Danish chiropractic practice.

## Methods

### Study design and framework

This manuscript describes the development of national recommendations for the history taking, examination, and clinical management of infants aged 0–12 months in chiropractic practice. The recommendations were commissioned by the Danish Society of Chiropractic and were initially developed in 2020 before being updated in 2025. The reporting of this manuscript was informed by the RIGHT Statement to improve transparency and completeness of reporting [7] (Supplementary file 1).

### Setting

The recommendations were developed for use in Danish chiropractic practice. In 2018, infants aged 0–12 months represented approximately 3.5% of all patients seen by Danish chiropractors, corresponding to approximately 13,000 consultations annually.

### National knowledge-sharing workshop

A national workshop on infant chiropractic care was organised by the Danish Society of Chiropractic and attended by 71 chiropractors with a special clinical interest in paediatrics.

The workshop consisted of invited presentations followed by facilitated small-group discussions and plenary sessions. Participants were encouraged to discuss clinical experiences, identify important elements of history taking and examination, highlight areas of uncertainty, and suggest additional considerations relevant to clinical practice. The purpose of the workshop was to gather practice-based knowledge and clinical perspectives rather than to achieve formal consensus.

Notes from all discussion groups were synthesised by the authors and circulated to all participants following the workshop to verify that the discussions had been accurately represented and to allow additional comments. No further comments were received. The verified workshop summary informed subsequent revisions of the recommendations.

### Literature review

The initial recommendations were developed through a combination of existing clinical expertise and a targeted review of the scientific literature. A literature search was conducted in PubMed without restrictions on publication year to identify evidence relevant to infants aged 0–12 months. The review focused on studies relating to medical history taking, physical and musculoskeletal examination, red flags, referral, and clinical management relevant to chiropractic practice.

The purpose of the literature review was to identify and summarise the available evidence to inform the recommendations rather than to undertake a formal systematic review. Studies were included if they informed one or more aspects of history taking, examination, referral, or management of infants relevant to chiropractic practice. No formal exclusion criteria were predefined because the purpose was to identify literature relevant to recommendation development rather than undertake a systematic evidence synthesis. The references cited throughout the manuscript represent the principal evidence sources underpinning the recommendations.

Recommendations concerning general medical history taking and physical examination were based primarily on a Danish paediatric medical textbook [5] and were considered alongside previously published international consensus recommendations describing best practice for chiropractic care of children [3]. Recommendations relating to musculoskeletal assessment and management were developed through the subsequent expert review process.

### Expert review and recommendation development

Following the workshop, an expert panel consisting of seven Danish chiropractors with extensive experience in paediatric chiropractic practice was convened. The panel had a mean age of 42 years (range 30–65 years) and an average of approximately 20 years of clinical experience. Panel members represented different regions of Denmark and had expertise in paediatric chiropractic practice or paediatric musculoskeletal research.

The expert panel did not develop the recommendations de novo. Instead, panel members critically reviewed successive drafts of the recommendations throughout the development process. During the first review round, panel members were asked to evaluate the overall structure, scope, completeness, and clinical relevance of the recommendations. Following revision, a second review round focused on section-specific feedback, clarity of wording, and clinical applicability.

Feedback was provided in both written and verbal form. All comments from the expert panel and the national workshop were reviewed jointly by the first and senior author. The first and senior author jointly reviewed all feedback received throughout the development process. Comments were discussed and incorporated where they improved clarity, clinical relevance, or consistency with the overall purpose of the recommendations. Final editorial decisions were made by the first author.

### External consultation

The revised draft recommendations were distributed by the Danish Society of Chiropractic for external consultation to relevant professional and healthcare organisations, including the Danish Chiropractic Association, the Danish Health Authority, the Danish College of General Practitioners, the Danish Midwives Association, the Danish Society of Health Visitors, and the Danish Paediatric Society.

Stakeholders were invited to provide written feedback on the content, relevance, and applicability of the recommendations. All comments received during the consultation were reviewed jointly by the first and senior author and considered for incorporation into the final recommendations. Revisions were made where comments improved clarity, accuracy, or clinical applicability while maintaining the overall scope and purpose of the recommendations.

## Results

The development process resulted in a set of national consensus-based recommendations covering five main areas: medical history, general physical examination, musculoskeletal examination, recognition of red flags and referral, and management considerations following assessment. The minimum and supplementary assessment components are summarised in Tables 1–6, followed by overarching management principles and considerations for common clinical presentations.

**Table 1:** Medical history minimum requirements regarding a child aged 0–12 months

General medical history information	Description of symptoms
	Primary and secondary complaint(s)
	Time of onset
	Known cause/trigger (if any)
	24-h variation
	Course progress—worse, no change, improved
	Improving/worsening factors
	Examined/treated by other health care provider
	Medicine consumption (incl. mother’s consumption when breastfeeding)
	Traumas
	Quiet/irritable/changed behaviour
General welfare	Growth (does the child grow as expected)
	Appetite
	Nutrition—Breastfeeding, infant formulae, solid food
	Sleep pattern—duration, interruption
	Crying pattern
	Bowel movements—diarrhoea/constipation/frequency
Pregnancy	Course
	Complications
Birth*	Course
	Vaginal—any use of vacuum—hard or soft cup- possible haematoma
	Caesarean section—immediate vs. planned—known presentation
	Child’s position at birth—normal, stargazing, seated, arm by head etc
	Child’s condition after birth
	Weight and length (see weight curves at [32])

^*^Parents might have an inaccurate or even misunderstood knowledge of exact obstetrical issues, like the birth presentation of the child. If there is a need for precise information about this, information from the hospital record is more comprehensive

### Medical history (anamnesis)

Parents know their child better than anyone and their observations and concerns should therefore be taken seriously. During history taking, the chiropractor should observe the infant’s general condition, including whether the infant is awake, attentive, responsive, and visually engaged. The chiropractor should sit at eye level with the parent, with the infant preferably held by the parent or seated on the parent’s lap [12].

The medical history for an infant aged 0–12 months should include, as a minimum, the domains listed in Table 1. Parents should be informed that questions about pregnancy, birth, feeding, sleep, crying, development, and general welfare are asked to understand the overall context for the consultation, and not necessarily to imply a causal relationship between these factors and the infant’s symptoms. Additional history-taking items should be included when clinically relevant, as showed in Table 2.

**Table 2:** Additional questions on the medical history of the child aged 0–12 months

Information about general medical history	Family diseases, e.g. allergies, asthma
	Vaccinations
General well being	Nutrition
	Breast—does the child latch properly, does it rest
	equally well with both breasts
	Infant formulae—type, has the type been changed, the child’s reaction to this
	Solid food
	Burps/vomit/hiccups
Course of the pregnancy	The mother’s use of medicine, tobacco and alcohol
Additional information	Side preferences, body asymmetries
	Motor skills development/milestones
	Siblings

### General examination

The first part of the physical examination should focus on the infant’s general health and on identifying signs of serious illness or other findings requiring referral. The examination should be explained to the parents in clear and understandable language, and parents should be invited to assist where appropriate. Instruments, such as a stethoscope, should be warm before use, and the most uncomfortable parts of the examination should be left until last. The infant should be undressed sufficiently to allow a thorough examination, while ensuring that the child does not become cold [12].

The chiropractor should allow time to comfort the infant when necessary, while avoiding an unnecessarily prolonged examination. Minimum requirements for the general examination of infants aged 0–12 months are shown in Table 3.

**Table 3:** Minimum requirements for an impartial examination of a child aged 0–12 months. Referral must be considered in the event of abnormal findings

**General observation**	Eye contact and alertness
	Spontaneous movement of body and extremities
	Temperature and respiration* (subjectively assessed)
	Age-dependent movement patterns
	Head control (3 months)
	Rolling (5–6 months)
	Crawling (8–10 months)
	Possible walking (10–16 months)
	Bruises, oedema etc., which may indicate’Battered child syndrome’
	Palpation of fontanels for abnormalities (level surface, fusion, pulse etc.)
**Other examination**	Palpation of clavicle for possible fractures and potential consequences of these
	General examination of muscles and joints—tonus and mobility
	Response to sound and light
	Primitive reflexes
	Babinski (0–18 months)
	Grasp reflex, palmar (0–4 months) and plantar (0 months until walking)
	Stepping reflex (0–2 months)

^*^Guiding values for respiration (breathing/minute) [33]

Birth: 25–60; 0–6 months: 25–55; 6–12 months: 20–40

### Musculoskeletal examination

When the history and general examination do not indicate serious illness or other findings requiring urgent referral, the chiropractor may proceed to a more detailed musculoskeletal examination. This part of the examination should assess movement, tone, symmetry, and age-appropriate neuromusculoskeletal function.

The minimum requirements for examination of the musculoskeletal system are presented in Table 4. Additional examination procedures may be included based on the infant’s presentation, parental concerns, and clinical suspicion, as outlined in Table 5.

**Table 4:** Standard requirements for objective examination of the musculoskeletal system in a child aged 0–12 months

Musculoskeletal	Examination of movement of the muscles and peripheral joints
	Examination of movement of the pelvis and columna
	Primitive reflexes (dependent on age)
	Sucking and search reflexes (0–4 months)
	Moro (0–4 months)
	Galant reflex (0–4 months)

**Table 5:** Additional general examination of the musculoskeletal system of a child aged 0–12 months [34]

Hip dysplasia	Observation of lines/wrinkles in buttocks and thighs
	Galeazzi
	Heel-to-buttock
	Difference in leg length
Breastfeeding difficulties	Palpation of jaws
	Palpation of os hyoideum
	Palpation/observation of tight frenum
Other	Palpation of cranium: symmetry, size of fontanels
	Deep tendon reflexes (depending on age and relevance)
	Primitive reflexes (depending on age)
	Landau (3–12 months)
	Parachute reflex (from 6–7 months)

### Red flags and referral

The history and examination should be used to identify signs of serious illness, developmental concerns, trauma, safeguarding concerns, or other findings requiring referral or interdisciplinary collaboration. Fever in an infant younger than three months, defined as a rectal temperature of 38.0 °C or higher, should be regarded as a potential sign of serious illness and requires immediate assessment by the child’s general practitioner, emergency department, or other appropriate medical service [10].

Older infants with signs of serious illness should also be referred, as outlined in Table 6. Febrile infants should be assessed for petechiae, neck or back stiffness, and, where relevant, a tense fontanel. Important differential diagnoses in febrile infants include airway infections, otitis media, childhood viral illnesses, gastroenteritis, appendicitis, urinary tract infections, meningitis, and encephalitis.

**Table 6:** (Based on Saxtrup 2019 [1])

Use your clinical eye: Is the child seriously ill?		
Behavioural signs	Signs of mild illness	Signs of serious illness
	Can be comforted	Irritable, whimpering
	Is awake or easily woken	Limp and not resisting
	Has good contact with its parents	Poor contact
	Reacts observantly	Lack of attention
Physiological signs	Good colouring	Pale, greyish colour
	Not dehydrated	Dehydrated
	Unobstructed breathing	Affected respiration and pulse

In infants, viral infections may be accompanied by a finely spotted rash. However, meningococcal disease is an important differential diagnosis in infants presenting with fever or rash. In cases of possible meningitis or encephalitis, symptoms and objective findings may be non-specific. The chiropractor should therefore pay close attention to parental concerns about even subtle changes in the infant’s behaviour, including whether the child is apathetic, unusually drowsy, irritable, difficult to comfort, vomiting, convulsing, or presenting with rash, petechiae, or other skin bleeding.

Petechiae are small bleedings in the skin that do not disappear under pressure. In meningococcal disease, petechiae are often bluish-red, irregular in shape, variable in size, and may be distributed across the face, trunk, and extremities. Viral illness may also be associated with small pink petechiae, particularly above the nipple line and following coughing or vomiting; if the infant is otherwise well, this is less suggestive of meningococcal disease.

If inflicted harm or neglect is suspected, the chiropractor must act in accordance with national safeguarding legislation and inform the appropriate authorities.

### Management considerations following assessment

#### Information and counselling

Information and counselling are central components of the chiropractic consultation. The content of counselling should be guided by the infant’s presentation, the findings from the history and examination, and the level of parental concern. Counselling may include advice about positioning, movement, motor development, handling, and exercises. A broader health perspective should also be considered where relevant, including advice consistent with public health recommendations.

#### Manual care

Manual care refers to treatment administered with the hands and may be directed towards muscles, joints, or movement function. In infants, any manual approach must be adapted to the infant’s age, size, developmental stage, clinical condition, and tolerance. Because the infant musculoskeletal system differs substantially from that of adults, any manual procedures must be considerably gentler than those used in adult care.

Manual care may include mobilisation, low-force techniques, or manipulation. Manipulation, or spinal manipulative therapy, is commonly defined as a high-velocity, low-amplitude impulse directed at a spinal segment, whereas mobilization refers to manual force applied within the passive range of motion without impulse or cavitation [11].

In infants, such procedures must be substantially modified. Based on studies of the tensile strength of the vertebral column in different age groups, it has been suggested that manipulation in infants younger than four weeks should use no more than 10% of the force used in adults, and up to 30% for infants aged 2–23 months [12].

#### Adverse events and safety considerations

Parents should be informed about possible adverse events to support informed consent and to reduce unnecessary concern following treatment. Existing reviews suggest that transient symptoms such as fatigue, irritability, local redness, or discomfort may occur, whereas serious complications appear to be very rare and are most often related to delayed diagnosis rather than the manual intervention itself [6, 13–15].

This reinforces the importance of careful history taking, thorough examination, and appropriate referral when serious pathology is suspected.

Data from compensation claims in Denmark and Norway from 2004 to 2012, and from Australian insurance data up to July 2019, did not identify compensation claims involving children in chiropractic practice [16, 20].

### General management principles

Current evidence for manual treatment of infants remains limited, particularly for specific infant complaints. Clinical decisions should therefore be based on evidence-informed practice, integrating the best available evidence, clinical expertise, the findings from the individual assessment, parental preferences, and careful monitoring of response to care [6].

Where there are clinical indications for manual care, no signs of serious disease or anomaly, and parents have received sufficient information to provide informed consent, a time-limited trial of care may be considered. The infant should be monitored closely for response and possible adverse events. If symptoms or function do not improve within a short period, generally within two weeks unless specific circumstances justify a longer period, the management strategy should be reconsidered and referral or interdisciplinary collaboration should be considered.

Communication with the infant’s general practitioner or other healthcare providers is particularly important when serious pathology is suspected, when the infant has been referred by another healthcare provider, or when care is concluded with a recommendation for further assessment or management elsewhere.

### Common clinical presentations

The following sections illustrate how the recommendations may be applied in three common presentations encountered in chiropractic practice. They are intended as examples of the clinical reasoning underpinning the recommendations rather than comprehensive evidence reviews or condition-specific management guidelines.

### Infantile colic

Infantile colic is one of the common reasons parents seek chiropractic care for infants. It is defined by an abnormal crying pattern and may have several underlying or contributing causes. The assessment should therefore focus on identifying signs of serious illness, feeding problems, cow’s milk protein allergy, parent-infant interaction difficulties, and possible musculoskeletal findings that may be relevant to the infant’s discomfort.

The evidence for manual treatment of infantile colic remains uncertain. Controlled studies have reported mixed findings, and the available studies are generally limited by small sample sizes and challenges related to blinding [17–21]. A trial of manual care may be considered only when the history and examination indicate a possible musculoskeletal contribution, there are no signs of serious disease or anomaly, and the parents have been appropriately informed.

Assessment should also include attention to the interaction between the infant and parents, as difficulties in interpreting infant cues may contribute to crying and parental distress [22]. Where relevant, collaboration with health visitors, general practitioners, or other healthcare professionals should be initiated. If cow’s milk protein allergy is suspected, further assessment and management should be undertaken in collaboration with the general practitioner or health visitor [24–26]. Probiotics may be considered as an alternative or supplement where symptoms appear related to the gastrointestinal system, although the evidence remains limited and heterogeneous [17, 19, 27].

### Asymmetry

Parents may seek chiropractic care because of asymmetry, such as a preferred side when sleeping or breastfeeding, reduced cervical range of motion, or positional cranial asymmetry. The assessment should focus on identifying whether the asymmetry reflects normal variation, positional preference, musculoskeletal restriction, developmental concerns, or signs requiring referral.

There is limited direct evidence regarding manual care for infant asymmetry. However, clinical assessment may identify reduced movement or functional asymmetry where advice, exercises, positioning strategies, or a time-limited trial of manual care may be considered. Manual care should only be considered when the history and examination indicate a possible musculoskeletal contribution and there are no signs of serious disease or anomaly. Parents should be counselled about positioning and exercises to counteract positional cranial deformation where relevant [28, 29].

#### Breastfeeding difficulties

Breastfeeding difficulties may have multiple causes and often require timely assessment to avoid early cessation of breastfeeding. Chiropractic assessment should focus on whether musculoskeletal findings, such as reduced cervical movement, jaw tension, or functional asymmetry, may contribute to difficulty latching or feeding. At the same time, the chiropractor should consider whether referral to, or collaboration with, appropriately trained breastfeeding professionals is indicated.

There is limited controlled evidence regarding manual care for breastfeeding difficulties. Case series have reported improvements in latch following manual therapy, but stronger evidence is lacking [30]. A time-limited trial of care may be considered when the history and examination indicate a possible musculoskeletal contribution, there are no signs of serious disease or anomaly, and parents are appropriately informed. If there are no musculoskeletal findings, or if there is no rapid improvement within approximately one week, the mother and infant should be referred to a qualified breastfeeding advisor, such as a midwife, health visitor, or International Board Certified Lactation Consultant, particularly if breastfeeding is at risk [31].

## Discussion

This manuscript describes the development of the first national recommendations for history taking, examination, and clinical management considerations for infants in Danish chiropractic practice. The recommendations were developed in response to the absence of practical guidance specifically addressing the assessment of infants in chiropractic care and aim to promote a structured, transparent, and consistent approach to clinical assessment and management. By defining minimum requirements for history taking, physical examination, recognition of red flags, and referral, the recommendations seek to support patient safety while facilitating communication with parents and other healthcare professionals.

### Comparison with existing frameworks and literature

Previous publications have described the characteristics of children presenting to chiropractic practice and the most common reasons for consultation [1, 3, 4]. Broader best-practice recommendations for paediatric chiropractic care have also been published through international expert consensus [6, 7]. More recently, standardized paediatric history and examination forms have been published to support structured clinical assessment in chiropractic practice [21]. However, to our knowledge, no previous publication has specifically described the development of practical recommendations outlining the minimum requirements for history taking, physical examination, recognition of red flags, referral, and subsequent clinical management for infants in chiropractic practice.

Although these recommendations were developed for the Danish healthcare system, the development process may also be relevant internationally. Many countries face similar challenges, where clinicians must integrate limited research evidence with clinical expertise when managing infants presenting with non-specific complaints. Transparent reporting of recommendation development may therefore facilitate adaptation to other healthcare systems while allowing recommendations to be modified according to local legislation, referral pathways, and professional scope of practice.

### Strengths and limitations

Several methodological strengths support the credibility of these recommendations. First, the recommendations were informed by multiple complementary sources, including a targeted literature review, structured practice-based input from a national workshop involving 71 chiropractors with a special interest in paediatric care, expert review by seven experienced paediatric chiropractors, and external consultation with relevant professional and healthcare organisations. Together, these components ensured that the recommendations were informed by both available evidence and extensive clinical experience. Second, the development process included several iterative review stages, allowing recommendations to be refined following workshop discussions, expert review, and stakeholder consultation. This iterative approach strengthened the clinical relevance, clarity, and feasibility of the final recommendations. Finally, the manuscript has been reported with consideration of the RIGHT Statement [11], improving transparency in describing the recommendation development process and facilitating future adaptation or updating.

Several limitations should also be acknowledged. First, the literature review informing the recommendations was targeted rather than systematic. Consequently, although relevant evidence was identified and incorporated, it is possible that some studies were not identified. Second, the evidence supporting chiropractic assessment and management of infants remains limited. For several recommendations, particularly those relating to clinical management, evidence from high-quality clinical trials is sparse [8–10]. Clinical expertise therefore necessarily played an important role in the development process. Third, although expert review and external consultation were used to refine the recommendations, no formal consensus methodology, such as a Delphi process or nominal group technique, was employed. Consequently, these recommendations should be viewed as expert-informed recommendations rather than formal consensus guidelines. Finally, the recommendations were developed within the Danish healthcare system and therefore reflect Danish legislation, referral pathways, and professional responsibilities. Adaptation may therefore be required before implementation in other healthcare settings.

### Implications for clinical practice

These recommendations provide chiropractors with a structured framework for the assessment of infants presenting to chiropractic practice. By defining minimum standards for history taking, physical examination, recognition of red flags, referral, and subsequent clinical management, they may contribute to greater consistency in clinical practice while supporting early recognition of infants requiring medical referral. The recommendations may also facilitate communication between chiropractors, parents, and other healthcare professionals by providing a transparent description of the clinical assessment process.

### Implications for future research

Future research should evaluate the implementation and uptake of these recommendations within clinical practice and explore their influence on clinical decision-making, referral behaviour, interprofessional collaboration, and parent experiences. As the evidence base continues to develop, future updates should incorporate emerging evidence, particularly regarding the effectiveness and safety of manual care for common infant presentations such as infantile colic, asymmetry, and breastfeeding difficulties. Similar recommendation development processes may also be undertaken in other countries to facilitate context-specific adaptation while improving consistency in paediatric chiropractic practice.

## Conclusion

These national recommendations establish minimum requirements for history taking, examination, and clinical management considerations for infants in chiropractic practice. Developed through a structured process integrating a targeted literature review, practice-based input, expert review, and external stakeholder consultation, they provide a transparent and practical framework to support safe, consistent, and collaborative paediatric care. The transparent reporting of the development process may also facilitate the adaptation and development of similar recommendations in other healthcare settings.

## Supplementary Information

Below is the link to the electronic supplementary material.

Supplementary Material 1

## Data Availability

Not applicable.

## Abbreviations

°C: Degrees Celsius
GP: General practitioner
IBCLC: International board-certified lactation consultant
PICO: Patient/problem, intervention, comparison, outcome
RIGHT: Reporting items for practice guidelines in healthcare
WHO: World health organization

## References

[1] N Saxtrup 2019 Subjektivt & objektivt: anamnese, undersøgelse og journal 4. udgave, 1. oplag Munksgaard København

[2] C Hawk MJ Schneider S Vallone EG Hewitt 2016 Best practices for chiropractic care of children: a consensus update J Manipulative Physiol Ther 39 3 158 168 10.1016/j.jmpt.2016.01.003 27040034

[3] L Hestbaek A Jørgensen J Hartvigsen 2009 A description of children and adolescents in Danish chiropractic practice: results from a nationwide survey J Manipulative Physiol Ther 32 8 607 615 10.1016/j.jmpt.2009.09.004 19836596

[4] A Allen-Unhammer FJH Wilson L Hestbaek 2016 Children and adolescents presenting to chiropractors in Norway: National Health Insurance data and a detailed survey Chiropr Man Ther 24 1 29 10.1186/s12998-016-0118-9 PMC4968013 27482377

[5] AM Marchand 2012 Chiropractic care of children from birth to adolescence and classification of reported conditions: an internet cross-sectional survey of 956 European chiropractors J Manipulative Physiol Ther 35 5 372 380 10.1016/j.jmpt.2012.04.009 22627100

[6] G Keating L Amorin-Woods 2023 Commentary on the 2019 Safer Care Victoria review Chiropr J Aust 50 1 52

[7] Y Chen K Yang A Marušic 2017 A reporting tool for practice guidelines in health care: the RIGHT statement Ann Intern Med 166 2 128 132 10.7326/M16-1565 27893062

[8] MC Brouwers ME Kho GP Browman 2010 AGREE II: advancing guideline development, reporting and evaluation in health care CMAJ 182 18 E839 E842 10.1503/cmaj.090449 PMC3001530 20603348

[9] Qaseem A, Forland F, Macbeth F, Ollenschläger G, Phillips S, van der Wees P; Board of Trustees of the Guidelines International Network. Guidelines International Network: toward international standards for clinical practice guidelines. *Ann Intern Med*. 2012;156(7):525–31 10.7326/0003-4819-156-7-201204030-00009 22473437

[10] Fever Symptoms & Treatment (for Parents) [Internet]. [cited 2025 Apr 16]. Available from: https://kidshealth.org/en/parents/fever.html

[11] G Bronfort M Haas RL Evans LM Bouter 2004 Efficacy of spinal manipulation and mobilization for low back pain and neck pain: a systematic review and best evidence synthesis Spine J 4 3 335 356 10.1016/j.spinee.2003.12.003 15125860

[12] AM Marchand 2015 A proposed model with possible implications for safety and technique adaptations for chiropractic spinal manipulative therapy for infants and children J Manipulative Physiol Ther 38 9 713 726 10.1016/j.jmpt.2015.09.001 23845198

[13] S Vohra BC Johnston K Cramer K Humphreys 2007 Adverse events associated with pediatric spinal manipulation: a systematic review Pediatrics 119 1 e275 e283 10.1542/peds.2006-1392 17178922

[14] F Driehuis TJ Hoogeboom MWG Nijhuis-van der Sanden RA De Bie JB Staal 2019 Spinal manual therapy in infants, children and adolescents: a systematic review and meta-analysis on treatment indication, technique and outcomes PLoS ONE 14 6 e0218940 10.1371/journal.pone.0218940 PMC6592551 31237917

[15] AJ Todd MT Carroll A Robinson EKL Mitchell 2015 Adverse events due to chiropractic and other manual therapies for infants and children: a review of the literature J Manipulative Physiol Ther 38 9 699 712 10.1016/j.jmpt.2015.08.008 25439034

[16] J Jevne J Hartvigsen HW Christensen 2014 Compensation claims for chiropractic in Denmark and Norway 2004–2012 Chiropr Man Ther 22 1 37 10.1186/s12998-014-0037-5 PMC4226888 25389462

[17] R Perry V Leach C Penfold P Davies 2019 An overview of systematic reviews of complementary and alternative therapies for infantile colic Syst Rev 8 1 271 10.1186/s13643-019-1185-2 PMC6844054 31711532

[18] LV Holm W Vach DE Jarbøl HW Christensen J Søndergaard L Hestbæk 2021 Identifying potential treatment effect modifiers of the effectiveness of chiropractic care to infants with colic through prespecified secondary analyses of a randomised controlled trial Chiropr Man Ther 29 1 16 10.1186/s12998-021-00364-7 PMC8054382 33874964

[19] SR Vaz MH Tofoli MAG Avelino PSS Costa Da 2024 Probiotics for infantile colic: Is there evidence beyond doubt? a meta-analysis and systematic review Acta Paediatr 113 2 170 182 10.1111/apa.16736 37962097

[20] D Carnes A Plunkett J Ellwood C Miles 2018 Manual therapy for unsettled, distressed and excessively crying infants: a systematic review and meta-analyses BMJ Open 8 1 e019040 10.1136/bmjopen-2017-019040 PMC5988120 29371279

[21] D Dobson PL Lucassen S Sampler 2003 Manipulative therapy for infantile colic Cochrane Database Syst Rev 4 CD004796 10.1002/14651858.CD004796 PMC11665957 23235617

[22] DM Zeifman J-R St 2017 Parenting the crying infant Curr Opin Psychol 15 149 154 10.1016/j.copsyc.2017.03.012 PMC5494986 28685155

[23] Underretningspligt [Internet]. [cited 2025 Apr 16]. Available from: https://www.sm.dk/arbejdsomraader/boern-og-unge-i-udsatte-positioner/underretningspligt

[24] DC Estep A Kulczycki 2000 Colic in breast-milk-fed infants: treatment by temporary substitution of neocate infant formula Acta Paediatr 89 7 795 802 10.1111/j.1651-2227.2000.tb00387.x 10943960

[25] I Jakobsson L Lothe D Ley MW Borschel 2000 Effectiveness of casein hydrolysate feedings in infants with colic Acta Paediatr 89 1 18 21 10.1111/j.1651-2227.2000.tb01190.x 10677051

[26] L Lothe T Lindberg 1989 Cow’s milk whey protein elicits symptoms of infantile colic in colicky formula-fed infants: a double-blind crossover study Pediatrics 83 2 262 266 10.1542/peds.83.2.262 2913556

[27] R Nocerino F Filippis De G Cecere A Marino M Micillo C Scala Di 2020 The therapeutic efficacy of *Bifidobacterium animalis* subsp. *lactis* BB-12® in infant colic: a randomised, double blind, placebo-controlled trial Aliment Pharmacol Ther. 51 1 110 20 10.1111/apt.15561 PMC6973258 31797399

[28] CJ Fludder BG Keil 2021 Deformational plagiocephaly and reduced cervical range of motion: a pediatric case series in a chiropractic clinic Altern Ther Health Med 27 6 26 32 32663178

[29] Skævt hoved hos spædbørn - Patienthåndbogen på sundhed.dk [Internet]. [cited 2025 Apr 16]. Available from: https://www.sundhed.dk/borger/patienthaandbogen/boern/sygdomme/knogler-og-led/skaevt-hoved-hos-spaedboern/

[30] C Parnell Prevost B Gleberzon B Carleo K Anderson M Cark KA Pohlman 2019 Manual therapy for the pediatric population: a systematic review BMC Complement Altern Med 19 1 60 10.1186/s12906-019-2433-x PMC6417069 30866915

[31] R Chowdhury S Khoury J Leroux R Alsayegh CM Lawlor ME Graham 2024 Alternative therapies for ankyloglossia-associated breastfeeding challenges: a systematic review Breastfeed Med 19 7 497 504 10.1089/bfm.2023.0269 38592282

[32] WHO Growth Charts [Internet]. [cited 2025 Apr 16]. Available from: https://www.cdc.gov/growthcharts/who-growth-charts.htm

[33] The Royal Children’s Hospital: The Royal Children’s Hospital [Internet]. [cited 2025 Apr 16]. Available from: https://www.rch.org.au/home/

[34] Pediatric Neurologic Examination Videos & Descriptions: 3 Month Old [Internet]. [cited 2025 Apr 16]. Available from: https://neurologicexam.med.utah.edu/pediatric/html/03month.html#11

